# The balanced mind: the variability of task-unrelated thoughts predicts error monitoring

**DOI:** 10.3389/fnhum.2013.00743

**Published:** 2013-11-07

**Authors:** Micah Allen, Jonathan Smallwood, Joanna Christensen, Daniel Gramm, Beinta Rasmussen, Christian Gaden Jensen, Andreas Roepstorff, Antoine Lutz

**Affiliations:** ^1^MindLAB, Aarhus University HospitalAarhus, Denmark; ^2^Department of Culture and Society, Interacting Minds Centre, Aarhus UniversityAarhus, Denmark; ^3^Department of Psychology, University of YorkYork, UK; ^4^Neurobiology Research Unit, Copenhagen University HospitalCopenhagen, Denmark; ^5^Department of Psychology, University of CopenhagenCopenhagen, Denmark; ^6^Brain Dynamics and Cognition Team, INSERM, CNRS, Lyon Neuroscience Research CenterLyon, France; ^7^Lyon Neuroscience Research Center, INSERM U1028, CNRS UMR5292, Lyon 1 UniversityLyon, France

**Keywords:** thought-sampling, response inhibition, error monitoring, mind-wandering, metacognition, variability, neurophenomenology

## Abstract

Self-generated thoughts unrelated to ongoing activities, also known as “mind-wandering,” make up a substantial portion of our daily lives. Reports of such task-unrelated thoughts (TUTs) predict both poor performance on demanding cognitive tasks and blood-oxygen-level-dependent (BOLD) activity in the default mode network (DMN). However, recent findings suggest that TUTs and the DMN can also facilitate metacognitive abilities and related behaviors. To further understand these relationships, we examined the influence of subjective intensity, ruminative quality, and variability of mind-wandering on response inhibition and monitoring, using the Error Awareness Task (EAT). We expected to replicate links between TUT and reduced inhibition, and explored whether variance in TUT would predict improved error monitoring, reflecting a capacity to balance between internal and external cognition. By analyzing BOLD responses to subjective probes and the EAT, we dissociated contributions of the DMN, executive, and salience networks to task performance. While both response inhibition and online TUT ratings modulated BOLD activity in the medial prefrontal cortex (mPFC) of the DMN, the former recruited a more dorsal area implying functional segregation. We further found that individual differences in mean TUTs strongly predicted EAT stop accuracy, while TUT variability specifically predicted levels of error awareness. Interestingly, we also observed co-activation of salience and default mode regions during error awareness, supporting a link between monitoring and TUTs. Altogether our results suggest that although TUT is detrimental to task performance, fluctuations in attention between self-generated and external task-related thought is a characteristic of individuals with greater metacognitive monitoring capacity. Achieving a balance between internally and externally oriented thought may thus aid individuals in optimizing their task performance.

## Introduction

Our day-to-day lives are rich with thoughts and feelings that emerge without a direct relationship to the here and now. So-called “task-unrelated thoughts” (TUTs) can be quite variable in content. We might think about our dinner plans while waiting for the bus, or rehearse an important speech in the shower. These self-generated experiences are unique insofar as they are not derived directly from an external stimulus; rather they form a train of endogenous thoughts, perceptually decoupled from ongoing sensory information and any task being performed (Smallwood, 2013). While such thoughts presumably facilitate goal-oriented behavior over longer time frames, they can also interfere with cognitive performance of tasks in the moment, for example when worrying about a negative social interaction causes us to forget to stop for groceries on the way home from work. An interesting and underexplored question is how TUTs both facilitate and interfere with behavior, and the underlying brain processes supporting these interactions.

Large-scale thought sampling studies investigating the context and intensity of TUTs suggest that self-generated thoughts may comprise a large part of our daily mental activity (Killingsworth and Gilbert, 2010) and have a complex relationship to psychological well-being, relating to both costs and benefits (Smallwood and Andrews-Hanna, 2013). For example, while increased TUT intensity is commonly reported in attention-deficit disorder and negative affect (Weyandt et al., 2003; Smallwood et al., 2007b; McVay et al., 2008; Marchetti et al., 2012), TUT-related benefits for cognition included creativity (Baird et al., 2012), an enhanced memory for personally relevant information (Smallwood et al., 2011), the opportunity to plan for the future (Baird et al., 2011), and a style of decision-making characterized by patience (Smallwood et al., 2013a). One important question in investigations of self-generated thought is therefore what determines whether for a given individual or context, TUT is associated with costs or benefits.

Investigating self-generated thoughts presents particular methodological difficulties, as their spontaneous nature renders direct experimental manipulation problematic. An established method used in the present investigation is to study the experience of TUTs while people perform an external task; an advantage of this approach is that the experiential reports can be validated by a process of triangulation using behavioral, physiological, and subjective measures recorded during the session (Jack and Roepstorff, 2002; Schooler, 2002). Neuroimaging studies have revealed that self-generated cognition is linked to functional activity in the posterior cingulate (pCC) and medial prefrontal cortex (mPFC), central hubs of the default mode network (DMN) (Mason et al., 2007; Christoff et al., 2009). The DMN is a constellation of cortical regions also including mPFC, pCC, and inferior parietal cortex (Greicius et al., 2003; Hampson et al., 2006; Andrews-Hanna et al., 2010; Anticevic et al., 2010) that reliably deactivates during cognitively demanding tasks.

While functional connectivity studies suggest that the DMN may be “anti-correlated” with the salience and control related networks (Fox et al., 2005; although see Murphy et al., 2009 for critique), the network also participates in a variety of functional processes important for self-regulation including prospection, episodic memory, and social cognition (Buckner and Carroll, 2007). Several functional magnetic resonance imaging (fMRI) studies have implicated activity in the mPFC and the pCC specifically with self-reports of mind-wandering, including one study that found that when participants reported being more aware of their mind-wandering, executive and DMN nodes co-activate (Mason et al., 2007; Christoff et al., 2009; Stawarczyk et al., 2011). Correlation between the control and DMN is also observed during autobiographical planning (Spreng et al., 2010). The extent and nature of interactions between these networks and their contribution to the costs and benefits of TUTs is currently unclear.

Consistent with a general functional role of self-generated thought, the mPFC is typically activated when thinking about the self and when making judgments about others (Mitchell, 2009). In addition, the medial and rostral-lateral portion of the PFC are also implicated in social cognition and metacognitive problem solving (Burgess et al., 2007; Dumontheil et al., 2010a,b) and individual differences in the volume of the rostral-lateral PFC predict metacognitive ability (Fleming et al., 2010). Metacognition supports flexible problem solving, and is thought to both monitor and control internal and external attention (Flavell, 1979; Fleming et al., 2012). Consistent with this notion, a recent resting state study by Baird et al. (2013) demonstrated that the medial section of the frontal pole shows increased functional integration with regions of the DMN for individuals with greater metacognitive performance on a memory task, while lateral regions of the mPFC predicted improved metacognition of perceptual processes. Together evidence for both self-generated thought and metacognition converge on the notion that the mPFC supports such processes, including those necessary to navigate complex social interactions (Amodio and Frith, 2006; Frith and Frith, 2012). Metacognitive monitoring enables an individual to correct problems in task performance, facilitating flexible responses. As such, a key aim of the current experiment was to both replicate the well-documented impairment of task performance by TUTs and also establish whether the monitoring of concurrent performance might be similarly impaired.

Error monitoring in the context of response inhibition is an extensively researched metacognitive ability, beginning with early work suggesting that correction of errors can occur as early as 200 ms post-error, before conscious awareness of having committed a mistake (Rabbitt, 1966, 2002). Although a variety of experimental methods exist for eliciting awareness of errors, a common difficulty relates to eliciting sufficient aware and unaware errors for comparison, due to an inverse relationship between task difficulty and awareness (e.g., participants typically make few errors with high overall awareness on easy tasks, or many errors with little awareness on difficult tasks). One effective experimental paradigm is the Error Awareness Task (EAT) in which a participant must inhibit their responses according to two competing stimulus rules (Hester et al., 2005, 2009, 2012; O'Connell et al., 2009). Prior studies have shown that awareness of errors in the EAT depends upon a distributed neural network including anterior insula, cingulate cortices, and medial frontal gyrus (Hester et al., 2005, 2012; Ullsperger et al., 2010). Here we used the EAT to distinguish the contributions of particular neural systems, including the salience and DMNs, to task performance, error monitoring, and TUTs. Additionally, we explored whether particular aspects of mind-wandering, such as its intensity and variability, would predict error monitoring performance.

To measure the experience of TUT we embedded experience sampling probes within the EAT, prompting participants to rate the subjective intensity of TUTs in the preceding interval. Previous investigations have utilized this approach in both behavioral (Mrazek et al., 2012) and neuroimaging (Christoff et al., 2009) research. Although it is unclear whether TUTs should be treated as a continuous or dichotomous state (although see Schad et al., 2012 for evidence of the former), our approach allows the estimation of the intensity of subjective experience within a given period. This offers the advantage of a metric that combines both the temporal occurrence and subjective intensity of different aspects of mind-wandering. We also explored a phenomenological distinction concerning the self-absorbing nature of TUTs. To do so, participants were trained to rate both the intensity of TUTs and the subjective “stickiness” of these experiences. We defined TUT stickiness as recurring thoughts that absorb attention or meta-awareness beyond their intrinsic frequency. Our aim was to dissociate the intensity of TUTs from their ability to absorb awareness, leading to rumination.

In addition to examining overall TUT and stickiness rates, we were also interested in the variability of these experiences. Within-subject variability reflects important dynamical aspects of cognition and experience, reflecting discrete state transitions (Varela et al., 2001; Lutz et al., 2002). While analysis of self-reported mean TUT yields information relating to the contents of introspective subjective awareness, we reasoned that variability in TUT report usage might reflect underlying trends in TUT experience not necessarily open to direct self-report. Previous research has demonstrated a link between reaction time variability during sustained attention tasks and attentional instability (Larson and Alderton, 1990; Stuss et al., 1994; Hultsch et al., 2002). Increased reaction time variability during sustained attention tasks is also predictive of psychopathology; people with ADHD typically show alterations on RT variance even when there are no discernable differences in mean RT (Leth-Steensen et al., 2000; Vaurio et al., 2009; Epstein et al., 2011). However, variability of reaction time can also be adaptive, for instance in slowing responses following error awareness (Shalgi et al., 2007), and variation in RT is reduced following intensive attention training (Lutz et al., 2009). Following commission errors participants rapidly decelerate motor responses (e.g., post-error slowing). This source of RT variability is thought to reflect flexible monitoring behavior, and is reduced both in patients with ADHD (Shiels et al., 2012) and following a negative mood induction—a period when the intensity of TUTs increase (Smallwood et al., 2009). As self-generated thought has both costs and benefits (Smallwood and Andrews-Hanna, 2013), it is conceivable that a highly variable style of thinking in which neither TUT nor task-related thought unduly dominate cognition could support flexible and adaptive cognitive performance.

In summary, the present experiment examined the relationship amongst within- and between-subject variability in TUT intensity and stickiness, response inhibition accuracy, and the awareness of consequent mistakes. Based on prior research, we expected increased TUT to be associated with worse inhibition performance and to engage prefrontal nodes of the DMN. The main focus of the experiment, however, was to ascertain the relationship between TUT and metacognition, which was assessed by measuring self-reported stickiness and error monitoring. One possibility is that better metacognition increases the ability to regulate mind-wandering (Schooler, 2002) and so for motivated participants under demanding conditions, the capacity to detect errors should correlate with reduced TUT. Alternatively, if TUT variability reflects flexible shifting between internal and external information, balancing both self-generated and perceptually directed thought, then we expected to find greater variability in TUT associated with increased error awareness.

## Methods

### Study participants

42 participants (27 females) were recruited from an online participant pool system in Aarhus, Denmark, from both the local university and community. The average age of participants was 34.8 years (±0.9 SEM, range = 25–47 years), with 17.6 mean years education (±0.5 SEM, range = 10–23 years). All procedures were approved by the local research ethics committee, De Videnskabsetiske Komitéer for Region Midtjylland, in accordance with the declaration of Helsinki. As part of a separate investigation concerning the impact of mindfulness on EAT and visual sensitivity, half of our participants (*n* = 21) were mindfulness meditation practitioners recruited locally using flyers and an online participant pool (Sona-Systems Experiment Management Software).

Here we were specifically interested in how individual differences in TUT experience and variability would predict EAT performance; inclusion of meditation practitioners in our sample was thus used as a strategy to maximize TUT-related variability within the sample. To ensure that our present findings were not biased by systematic group differences, all analyses were conducted using group status as a nuisance covariate. Specific group contrasts are not examined here; although they will be reported in a follow-up investigation of the impact of mindfulness training on EAT performance and visual sensitivity. Groups were matched for age (mean age meditation = 35.1 years, mean age control = 34.6 years), gender (meditation = 14 males, control = 15 males), and education (controls mean education = 16.5 years; meditation mean education = 18.6 years). In our meditation study we aimed to specifically sample “adept” practitioners; inclusion criteria specified that participants must practice at least 20 min per day at a minimum of 3 times per week over the two years prior to the study, and have attended at least 1 meditation retreat in the previous year (mean hours practiced = 1303.6).

All fMRI scans were acquired over a one-week period following enrollment in the study. Participation in the fMRI scan was incentivized with a 200DKK (approximately $35 USD) reimbursement, and to control motivation all participants were instructed that the top 1/3rd of scores on the scanning task would receive an additional 200DKK (Jensen et al., 2012).

### Experimental procedures

Before scanning, participants were informed that the purpose of the study was to investigate individual differences in their attentional ability. Participants visited the lab twice, once to provide informed consent and complete a psychophysical vision sensitivity test (data not reported here) and again to complete the fMRI scan. Specifically the psychophysics test was the “theory of visual attention task” (TAVT). This measure was included to replicate a previous result that meditation experience improves TAVT performance irrespective of motivation levels (Jensen et al., 2012) and is thus not analyzed here. Participants completed 6 runs of the EAT within the scanner, ~45 min in total. Immediately following the scan, participants completed a debriefing survey, rating (0–100) their experienced difficulty, interest in the task, task effort expended, and self-estimated stop accuracy and error awareness. These measures were included as part of the meditation study to investigate the role of perceived effort, interest, and retrospective metacognition in detected group differences. They are presented here as overall summary measures indicating general participant engagement with the task. See Table 1, below for descriptive statistics of these measures.

**Table 1:** **Correlations among study variables**.

	**TUT_Mean_**	**TUT_Variance_**	**Stick_Mean_**	**Stick_Variance_**	**SA**	**EA**
TUT_Mean_	–	0.269	0.784^**^	0.241	−0.561^**^	−0.128
TUT_Variance_		–	0.163	0.654^**^	0.098	0.417^**^
Stick_Mean_			–	0.401^*^	−0.436^**^	−0.127
Stick_Variance_				–	−0.069	0.299
SA					–	0.332^*^
EA						–

** *p < 0.01*,

* p <0.05. Correlation table for primary independent and dependent variables. Abbreviations: TUT_Mean_, mean task unrelated thought (reversed); TUT_Variance_, within-subject standard deviation of TUT; Stick_Mean_, mean stickiness rating; Stick_Variance_, within-subject standard deviation of stickiness; SA, stop accuracy; EA, error awareness.

### Behavioral measures

#### Error awareness fMRI task

**Figure 1:**
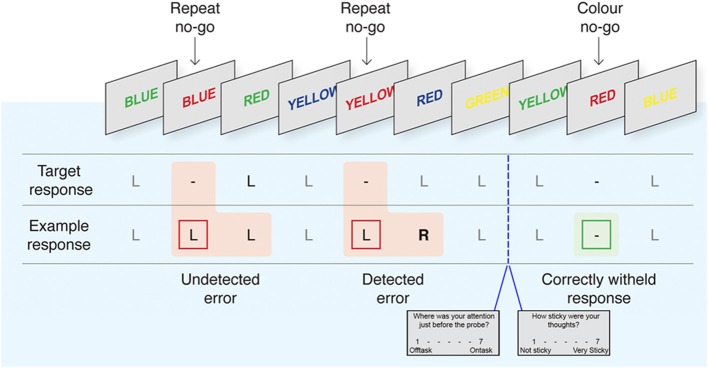
**The EAT task with interleaved thought probes**. Adapted with permission from Hester et al. (2012). Participants respond by pressing the left button (L) during Go trials and withhold from responding (−) to repeated or font-color matching words. Following commission errors, participants are trained to forgo the normal Go response to instead press the right button “R” indicating error awareness for that trial. Pseudo-randomly intermixed “thought probes” prompted participants to rate the intensity of TUTs and their “stickiness” in the pre-probe interval. See Methods for detailed overview of task timing and instructions.

To assess individual differences in response-inhibition and error monitoring, we adapted the delayed-response EAT from Hester et al. (2005, 2009, 2012) and Shalgi et al. (2007) (see Figure 1 for a task schematic). The EAT requires participants to respond to a serial presentation of color-words in incongruent font colors (i.e., the word “blue” colored *red*). Participants were instructed to respond to Go trials by pressing the “1” button on a 2-button box with the right index finger. No-go trials in which participants were instructed to withhold their response (“stop”) occurred on approximately 11% of the total trials, according to two stop rules, “repeat” and “color-match” (Hester et al., 2009, 2012). In the latter, participants were required to stop whenever a word was presented in matching font-color, and in the former whenever a word was repeated on two consecutive trials. Each trial consisted of a stimulus (600 ms) followed by an interstimulus interval (900 ms).

Participants completed 6 runs of the EAT within the scanner, each consisting of 200 Go and 25 pseudo-randomly intermixed Stop trials, for a total of 1350 trials. In order to maximize unaware errors and mind-wandering, participants were trained to respond during the interstimulus interval, emphasizing accuracy and timing consistency over absolute response speed, increasing the repetitive nature of the Go task as in Shalgi et al. (2007). Response timing has been used previously on the EAT task and typically reduces the intersubject variability of responses (Hester et al., 2005). In the case of a commission error, on the trial immediately following that error participants were instructed to forgo their normal Go response and to instead “fix” their error using a second button (right middle index finger), indicating error awareness. Participants were randomly asked throughout the task to answer probes regarding their experience during the task using the 1 (right index) and 2 (right middle index) button (see below). Each probe lasted up to 6 s in total followed by a fixation cross (duration = 6 s – probe duration).

Because the occurrence of TUTs is negatively related to task difficulty (Christoff et al., 2009), task-overlearning was promoted prior to scanning by training all participants on 2–4 practice runs of the EAT until a minimum of 40% stop and 40% error awareness rate was reached. To control participant's motivation to perform the task, participants were instructed that they would gain an additional 200 DKK (about 35 USD) if they were within the top 1/3rd of EAT performance. Participants were further reminded that “fixing” commission errors via the report button would cause those errors to not count against their total score, to ensure that participants did not selectively focus on stopping to the exclusion of error reports.

Individual stop accuracy and error awareness scores were determined for each participant. Stop accuracy was calculated as the ratio of correctly withheld stop trials over the total number of stop trials (Correct stops/total stops), and error awareness as the ratio of number of reported error trials over total number of error trials (aware errors/unaware errors). Go accuracy was calculated as the ratio of the total number of correct responses (e.g., reaction time > 0 in a Go trial) over the total number of Go trials, excluding trials following errors (which are confounded by the error reporting response). Mean TUT and stickiness scores, as well as within-subject standard deviations were calculated for each participant as measures of the average content and variance of each subjective dimension.

Prior to analysis participants with extremely low stop accuracy (indicating a failure to correctly perform the task) were identified; one participant with <50% accuracy was excluded from all subsequent EAT-related analyses. As overall performance was generally high, three participants had too few errors (<5) to be included in error related behavioral analyses and were excluded (O'Connell et al., 2009; Hester et al., 2012). Finally, for our error related fMRI analysis 2 additional participants with 0 aware errors were excluded. A total of 6 participants were thus excluded from the error related fMRI analysis.

#### Subjective mind-wandering reports

Subjective awareness of TUTs was assessed in a similar fashion to Christoff et al. (2009), using interleaved “thought probes” distributed pseudo-randomly throughout the EAT task. Each probe consisted of two questions, one evaluating the subjective intensity of TUTs, “Where was your attention focused just before the probe?” and the second evaluating the “sticky” or ruminative quality of mind-wandering, “How sticky were your TUTs just before the probe?.” Participants responded using a 7-point scale ranging from “completely offtask” to “completely ontask” for the first and “completely sticky” to “not at all sticky” for the second. As we were interested in investigating particular phenomenological properties of mind-wandering, we combined two previous approaches to sampling task irrelevant thoughts. First, to minimize differences in scale properties we utilized a TUT scale in which participants rated their pre-probe thoughts as “ontask” or “offtask” as in Christoff et al. (2009). However, to specifically operationalize the subjective intensity of mind-wandering, we adapted phenomenological descriptions of TUTs from Mason et al. (2007). Participants were thus instructed that being “ontask” specifically meant that they had a “low frequency of task irrelevant thoughts,” with task irrelevant thoughts being defined as “any that do not facilitate performance and are not immediate reactions to perceptual information gleaned over the course of a trial.” All participants indicated understanding that ratings on the ontask/offtask scale corresponded to this definition. Examples of task relevant thoughts were given, such as those concerning the color of a word on the previous trial.

By fixing this subjective dimension, we aimed to stress the possibility of dissociation between high intensity TUTs with little impact on participants' metacognitive capacity and high-intensity TUTs that fully absorbed the participants' attention (e.g., “sticky” TUTs). Participants were therefor instructed to rate the “stickiness” of their task-irrelevant thoughts, with sticky thoughts defined as those that “distract (the participant) for a greater period of time, and are more attention catching than other task-irrelevant thoughts; this experience is sometimes described as being ‘lost in thought’.” Stickiness was thus included to explore whether ruminative and absorptive TUT compared to non-ruminative and non-absorptive TUT differentially impact sustained attention and error awareness (Koster et al., 2011; Van Vugt et al., 2012). Examples were given to emphasize the decoupled nature of sticky and unsticky task-irrelevant thoughts; participants were instructed that for example certain thoughts might arise (“What am I having for dinner tonight?”) but be relatively non-distracting from the task, whereas others (“Did I leave my oven on?”) might recur frequently throughout the task and demand more attention. Participants were provided with further examples until they indicated a good understanding of the distinction and completed practice probes during the EAT training session. During scanning participants completed 26 probes in total, 4–5 per 6 EAT runs, with one probe event (“focus” and “sticky”) occurring pseudo-randomly every 40–60 trials throughout the EAT. Each probe appeared for a maximum of 6 s followed by a fixation cross lasting up to 6 s depending on probe reaction time (e.g., 6 - Probe Duration). Due to the dependent nature of sticky thoughts on having some TUTs, we did not counter-balance the order of focus and sticky. Stickiness was thus operationalized as a second-order judgment on the quality of those TUTs reported in the first probe.

#### Auxillary recordings

As the BOLD signal reflects complicated neurovascular coupling, a considerable portion of BOLD variability can be explained by non-neural origins such as respiratory and cardiovascular fluctuation (Glover et al., 2000; Lund et al., 2006). Previous investigations have shown that regions implicated in both error monitoring (e.g., insula and cingulate) and mind-wandering (mPFC) are among the most susceptible to such artifacts, with as much as 8% of event-related variance being explained by these sources (Chang and Glover, 2009; Chang et al., 2009). To exclude such confounds and improve overall signal-to-noise ratio, we recorded both respiration and pulse in parallel with EPI image acquisition, in order to apply a nuisance variable regression approach to modeling serial correlations in the BOLD time series (Lund et al., 2006). During the functional MRI acquisition the cardiac and respiratory cycles were recorded with an infrared pulse oximeter on the patient's index-finger and a pneumatic thoracic belt, respectively.

All pulse and respiration time series were visually examined for acquisition artifacts (e.g., clipping, drop-out). Due to technical failure of the respiration belt, respiration time series were severely confounded and discarded from further analysis. While inclusion of both respiratory and pulse regressors has been shown to provide an optimal estimation of serial correlation, inclusion of pulse and motion regressors without respiration has been shown to also outperform standard autoregressive (“AR1”) noise-whitening techniques, particularly at faster repetition times (e.g., TR <4 s) (Lund et al., 2006). Descriptive statistics (mean heartbeats per min) were calculated for each subject. One participant's physiological data were lost due to technical failure and was hence excluded from all fMRI analyses.

#### fMRI acquisition protocols and preprocessing

Echo-planar images (EPI) were acquired at the Aarhus University Hospital, using a T2^*^-weighted, gradient echo sequence on a 3 Tesla (Siemens Trio) scanner, equipped with a 32-channel head coil. EPI images were acquired in an interleaved slice acquisition order (*TR* = 2000 ms, *TE* = 30 ms, flip angle = 90°, 47 slices of 3 mm thickness, in-plane resolution of 3 × 3 × 3, FOV = 192 × 192 mm). Soft cushions were used to minimize head movement.

All fMRI preprocessing and data analyses were performed in SPM8 (version 4667) (Friston et al., 2006). Default settings were used throughout, unless otherwise specified. The functional images of each participant were realigned and resliced (Friston et al., 1995), spatially normalized to MNI space using the SPM EPI template and trilinear interpolation (Ashburner and Friston, 1999), and smoothed using a 8 mm full-width half-maximum (FWHM) smoothing kernel (Worsley and Friston, 1995; Friston et al., 2000). Serial correlations were modeled using a nuisance variable regression approach (Lund et al., 2006). In addition to the SPM8 standard discrete cosine set high pass filter (128 s cut off), this approach includes 10 regressors based on cardiac and/or respiratory oscillations (Glover et al., 2000) and 6 motion parameters obtained from the realignment algorithm (Friston et al., 1996).

### Analysis

#### Error awareness task—reaction times and accuracy

To compare our results with previous experiments using the EAT, we analyzed accuracy and reaction time values across conditions. Go reaction times for each participant were calculated as the mean of all correct Go trials, excluding responses 2 SD below the participants mean Go RT. For comparison to previous experiments with the EAT, stop accuracy, error awareness, and mean reaction times were calculated for each subcategory of stop, i.e., color and repeat stop accuracy, color and repeat aware/unaware errors, and RT to color and repeat stop errors. Mean reaction times where inspected for values ±2 *SD* from the mean, resulting in the removal of 3 participants RT data. Reaction times were entered into One-Way repeated measures ANOVAs, within subject factor Response type (Go, Aware, Unaware) to assess differences in response speed across condition. Finally, we analyzed Stop Accuracy and Error awareness for each error subcategory (Repeat, Color) in separate one-way repeated measures ANOVAs.

#### Mind-wandering and behavior

**Figure 2:**
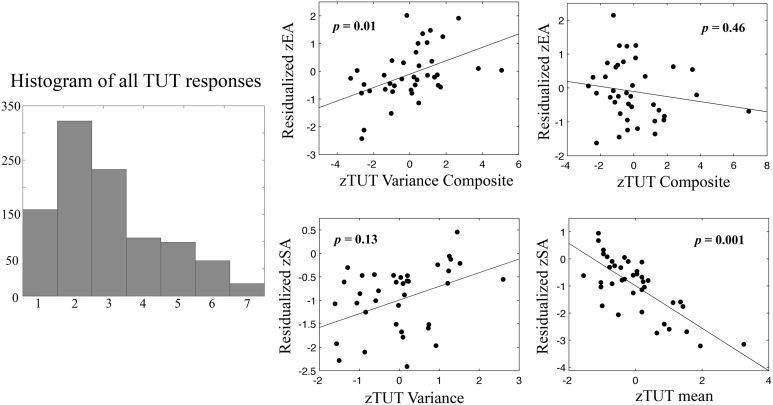
**Regression plots of TUT_mean_ TUT_Variance_ vs. error awareness (EA) and stop accuracy (SA)**. On the **left**, a histogram of all TUT responses recorded in the session showing continuous distribution of responses from 1 to 7 (scale response value on x-axis). Plotted relationships show a clear relationship between individual differences in TUT_Mean_ and stop accuracy, such that higher levels of TUT predict worse inhibition performance (bottom right). In contrast high levels of TUT_Variance_ predict increased error monitoring (**top left**). Scatterplots depict relationships between TUT_Mean_, within-subject standard deviation of TUT (TUT_Variance_), or composite mean measures (TUT_Mean_ + Stickiness_Mean_) and composite within-subject variance measures (TUT_Variance_ + Stickiness_Variance_). Prior to analysis TUT scores were reversed so that higher numbers reflect increased TUT, and all variables were transformed into *Z*-scores. Dependent variables are residualized for group status and opposing variables (e.g., SA for EA, TUT_Mean_ for TUT_Variance_, TUT composite for TUT variance composite, respectively). Data points represent individual participant scores. *p*-values show significance for each predictor variable from multiple regression model (see Results for more details).

Our first aim was to replicate previously reported relationships between performance and TUTs. To establish whether or not TUT ratings were utilized as a continuous or discrete measure, we created response histograms across all collected ratings, which showed a clear continuous distribution indicating that participants did not treat the scales as discrete binary measures (Figure 2). As TUT variance and stickiness had not been previously investigated, we then conducted a cross-correlation analysis to determine measurement colinearity. All scores were converted into *Z*-scores at the group level. Correlations between TUT_Mean_., TUT_Variance_, Stickiness_Mean_, Stickiness_Variance_, error awareness and stop accuracy were calculated (see Table 1). TUT_Mean_ and Stickiness_Mean_ were highly correlated (*r* = 0.78, *p* < 0.001). TUT_Mean_ also predicted stop accuracy (*r* = −0.56, *p* < 0.001). TUT_Variance_ was correlated with Stickiness_Variance_, (*r* = 0.65, *p* < 0.001) and EA (*r* = 0.42, *p* < 0.01). Stickiness_Mean_ correlated with Stickiness_Variance_ (*r* = 0.40, *p* < 0.03) and with stop accuracy (*r* = 0.44, *p* < 0.01). Finally, SA and EA showed moderate correlation (*r* = 0.33, *p* < 0.05).

#### fMRI analysis—single subject level

Following preprocessing, functional BOLD data were analyzed using an event-related hierarchical general linear modeling approach (Friston et al., 1994). Sessions were first concatenated and then entered into a first level design matrix modeling fixed linear effects over the entire time series. Each fMRI time series was modeled using 3 event-related regressors (duration = 0 s) for each condition of interest, in order: Correct Stop Trials, Unaware Errors, Aware Errors, as well as a separate TUT probe regressor (30 s duration epoch) shifted 37.50 s (25 trials) before each thought probe occurred, and a parametric modulation of the probe regressor encoding the rating for that probe. Due to the high correlation between TUT and stickiness ratings, we estimated two separate models, one with the mean TUT rating only and one with the stickiness only. Stop trials were modeled as the onset of each correct stop. Aware and unaware error trials were modeled as the onset of the trial in which the error occurred. In the EAT, Go trials are commonly left unmodeled as implicit baseline (Hester et al., 2005, 2012; O'Connell et al., 2009). The onset of each probe block regressor was jittered ± ~1 s. Probe regressors thus modeled task-related activity in the pre-probe interval and the linear modulatory effect of self-reported TUT intensity during that period. In addition to conditions of interest, each session model included 10 pulse and 6 motion-realignment nuisance regressors, to model confounding effects of these parameters. Session offsets modeled between-session variance. Fixed-effects of interest were identified using unidirectional *t*-contrasts for correct stop trials [(1 0 0 0 …)], aware > unaware errors [(0 −1 1 0…)], and linear correlation with the TUT report parameter [(0 0 0 0 1)]. As is common in error awareness paradigms, participants generally report substantially fewer errors than they commit (see Table 2 for summary of average total errors in each condition), leading to concerns that comparison of aware and unaware events may be unduly biased. Previous use of the EAT (Hester et al., 2005) has demonstrated that the aware vs. unaware analysis is not biased toward activity from aware errors.

**Table 2:** **EAT behavioral and post-scan summary measures**.

**Category**	***M***	***SD***
**ACCURACY AND ERRORS**		
Go accuracy (% correct)	80.3	0.1
No-go accuracy (% correct)	77.0	14.3
Repeat No-go accuracy	81.0	18.0
Color No-go accuracy	72.8	21.0
Error awareness (% of aware errors)	35.5	20.0
Repeat error awareness	26.9	20.6
Color error awareness^*^	43.7	24.4
Aware errors (total)	12.0	7.8
Unaware errors (total)	25.3	18.6
**REACTION TIMES (ms)**		
Go	1103.32	11.49
Aware error	1086.38	14.68
Unaware error^**^	1060.31	17.31
**SUBJECTIVE AND PHYSIOLOGICAL MEASURES**		
Task unrelated thought (1–7)	3.0	1.1
Stickiness (1–7)	2.5	1.0
Heartbeats per min	65.1	8.7
**POST-SCAN DEBRIEF (1–100)**		
Estimated EA	49.1	26.9
Estimate SA	76.8	14.0
Difficulty	56.0	23.3
Effort	92.5	9.1
Interest	63.2	28.3

* Significant difference between repeat and color error awareness p < 0.01.

** Significant difference between GoRT and unaware error RT, p < 0.01. Table shows mean (M) and standard deviation (SD) for accuracy of each EAT stop and error sub-category, as well as mean reaction times for Go, Aware, and Unaware Error trials. Mean self-reported task unrelated thoughts, reversed so higher numbers correspond with greater TUT, and post-scan responses to visual analog scale for self-estimated task performance, task difficulty, effort spent, and overall interest. See Methods for detailed task description.

#### fMRI analysis—group level

Our random-effects (RFX) analysis focused on three contrasts: correct stops (vs. baseline), aware vs. unaware errors, and the negative correlation of TUT reports and task-related BOLD activity. All RFX analyses were conducted by passing each participant's corresponding contrast image (stops, aware > unaware, TUT ratings) to a one-sample *t*-test. These contrasts were corrected for multiple comparisons using Gaussian random-field-theory, peak level family-wise error threshold (FWE) *p* < 0.05 (Worsley et al., 1996). For our analysis of BOLD correlation with task-unrelated thoughts, a mask of the DMN was created by conducting an automated meta-analysis on the Neurosynth database for “mPFC” (http://neurosynth.org) (Yarkoni et al., 2011). As the TUT parameter encoded the intensity of TUTs (1–7) prior to each probe, low values encoded higher levels of TUT. Thus, at the group contrast level, we tested for areas where greater reports of TUT predicted higher BOLD activity (negative correlation with TUT ratings and BOLD). To restrict the mask to primary clusters in mPFC, pCC, and inferior parietal lobes the downloaded NIFTII image was binarized at a *Z*-score > 4 threshold. The resulting mask (see Figure 5) was visually inspected to confirm that it provided good coverage of key DMN nodes, particularly in mPFC and pCC, and was subsequently applied in a region of interest analysis using the Wake Forest University (WFU) Pickatlas toolbox v3.0 (Maldjian et al., 2003, 2004), cluster-level corrected for multiple corrections, voxel selection threshold *p* = 0.01, pFWE <0.05 (Hayasaka et al., 2004). To ensure BOLD results were not biased by group status, all random effects contrasts included group status as a covariate of no-interest.

## Results

### EAT—behavioral analysis

Participants correctly withheld on the majority of stop trials (Mean *SA* = 77.0%, *SD* = 14.3%) and on average reported approximately 36% of total stop errors (Mean *EA* = 35.5%, *SD* = 0.20%). Stop accuracy was similar to previously reported results using the delayed-response EAT (*M* = 83.88, *SD* = 13.98), but with lower error awareness (*M* = 59.09, *SD* = 18.55), suggesting that task-overlearning successfully promoted automatic responding (Shalgi et al., 2007). Mauchly's test indicated that EAT reaction times violated the assumption of sphericity, χ^2^(5) = 8.54, *p* = 0.014, therefore the degrees of freedom were corrected using a Huynh-Feldt estimate of sphericity (ε = 0.85). There was a significant effect of response condition on reaction time (RT) *F*_(1.69, 57.77)_ = 5.17, *p* = 0.012; *post-hoc* comparisons demonstrated that this effect was driven by a significant difference between Go RT (*M* = 1103.32 ms, *SD* = 11.49 ms) and Unaware Error RTs (*M* = 1060.31 ms, *SD* = 17.31 ms), mean difference = −43.0 ms, *p* = 0.008. This decrease in RT during unaware errors is consistent with prior studies showing a link between task automaticity and mind-wandering (Smallwood et al., 2007a,2008b, 2011). Aware error RTs (*M* = 1086.38 ms, *SD* = 14.68) did not differ significantly from unaware or Go RTs. See Table 1 for a complete summary of EAT behavioral results. Participants showed a trend toward committing more errors during color than repeat trials *F*_(1, 40)_ = 3.97, *p* = 0.053, and they also reported being aware of more errors during color than during repeat trials *F*_(1, 38)_ = 14.32, *p* = 0.001.

### Mindwandering mean and variance vs. EAT behavior

Regression analysis with TUT_Variance_, TUT_Mean_, Stickiness_Variance_, and Stickiness_Mean_, group status, and error awareness as predictors explained 53.5% of stop accuracy variance [*F*_(6, 38)_ = 6.15, *p* < 0.001]; mean TUT was a highly significant predictor, β = −0.78, *p* = 0.001. Additionally group status significantly predicted SA, β = 0.33, *p* = 0.014. Follow-up *t*-tests revealed that the meditation group exhibited approximately 10% more stop accuracy (*p* = 0.022). No other predictors were significant. The same model with error awareness as the dependent variable explained 27.9% of the variance in error awareness at an above-threshold level [*F*_(6, 38)_ = 2.07, *p* = 0.085]; no individual predictors reached significance. Given the high level of correlation between TUT and stickiness for both variance (*r* = 0.65, *p* < 0.001) and mean (*r* = 0.78, *p* < 0.001), we constructed composite indices of mind-wandering variance (TUT_Variance_ + Stickiness_Variance_) and mean (TUT_Mean_ + Stickiness_Mean_) and repeated the regression analysis with the composites scores as predictors (again with the opposite DV and group status as control variables).

The resulting regression models explained 44.2% of stop accuracy variance [*F*_(4, 38)_ = 6.74, *p* < 0.001] and 27.4% of error awareness variance [*F*_(4, 38)_ = 3.21, *p* = 0.025], respectively. Within the model predicting stop accuracy, the mean mind-wandering composite was a significant predictor, β = −3.49, *p* = 0.001, as well as group status, β = 0.31, *p* = 0.026. Conversely, within the model predicting error awareness, only the mind-wandering variance composite significantly predicted EA, β = 0.44, *p* = 0.010. The observed difference between the inclusive and composite models suggests that TUT_Mean_-related variance (as opposed to Stickiness_Mean_) was the primary predictor for SA. Importantly, EA exhibited strong zero-order correlations with TUT_Variance_ and showed no correlation with Stickiness_Variance_. Thus, including stickiness in the overall model only reduced model sensitivity, as shown by the significant effect of the variance composite on EA, suggesting that only the unique TUT-related variance predicts EA (e.g., the only observed impact of modeling Stickiness_Variance_ can be explained by the reduced degrees of freedom for that model). We thus report the composite here for completeness, noting that the high multi-colinearity of the Stickiness and TUT variance likely reduces our ability to distinguish them in a regression model (Farrar and Glauber, 1967). These results suggest that individual differences in the average and variability of mind-wandering are specific predictors of EA and SA ability, with increasing absorption in internal thought predicting worse stop performance, and higher levels of variability predicting greater error awareness. See Figure 2 for plots of these relationships.

### fMRI—overall responses to stop and aware vs. unaware errors

**Figure 3:**
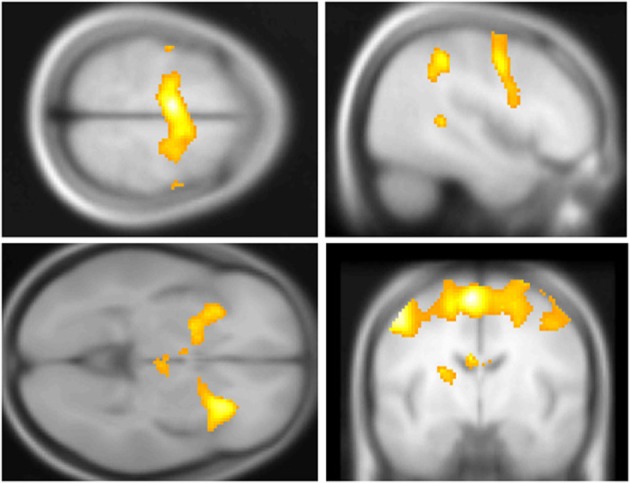
**Central executive and salience network BOLD responses to correct stop trials vs. baseline**. Significant activations throughout the motor control and salience networks, including premotor/supplementary motor area (**top left** and **right**), anterior insula (**bottom left**), putamen, (**bottom left**) and middle frontal gyrus (**top right**) BOLD activations to correct stop trials, shown in yellow. Voxel-wise statistical parametric maps (pFWE < 0.05, *k* threshold > 5 contiguous voxels) superimposed on SPM canonical anatomical image, average of 305 T1-weighted images. **Top left** shown at MNI Z = 62, top right at X = 47, **bottom left** at *Z* = −3, **bottom right** at *Y* = −8. See Table 3 for a complete list of foci.

**Figure 4:**
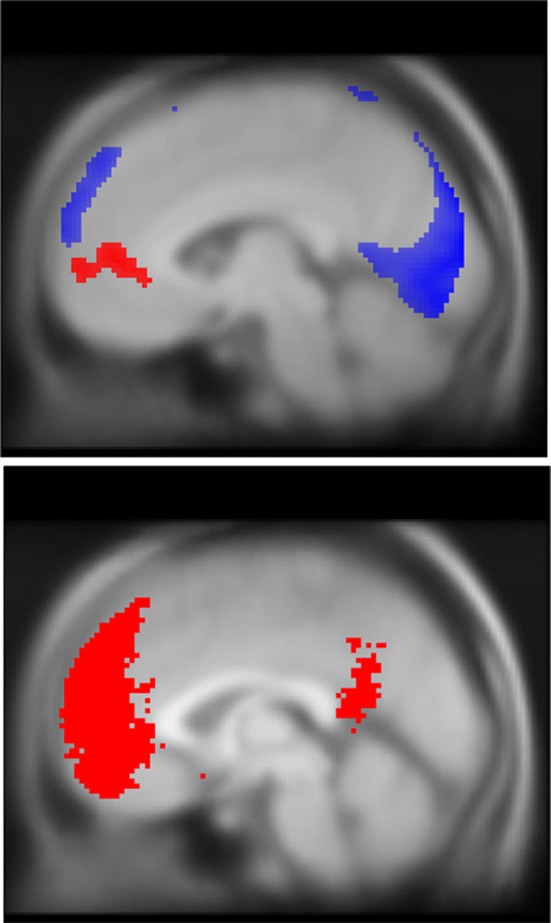
**DMN deactivations during stop trials (blue, top) and correlation with TUT reports (red, top), and mask used for ROI analysis (red, bottom)**. Note that while both task-unrelated thoughts and response inhibition engage the DMN, they recruit spatially unique regions of the mPFC. To facilitate comparison of spatial topography for EAT and TUT-related DMN activity, both activation maps are overlaid on a single MNI structural brain. In blue, significant deactivations during EAT stop trials (pFWE peak < 0.05, *k* threshold = 5 contiguous voxels). In red, increased self-reported TUT predicts greater mPFC BOLD activation, pFWE cluster < 0.05, region-of-interest analysis with DMN mask volume (bottom image shown in red), *k* threshold = 685 contiguous voxels. DMN mask generated using automated meta-analysis for term “mPFC” on neurosynth.org, *z*-score threshold > 4 (see Methods for further details). Statistical parametric maps superimposed on SPM canonical anatomical image, average of 305 T1-weighted images. Top image shown at MNI *X* = −5, bottom at *X* = 0.

**Figure 5:**
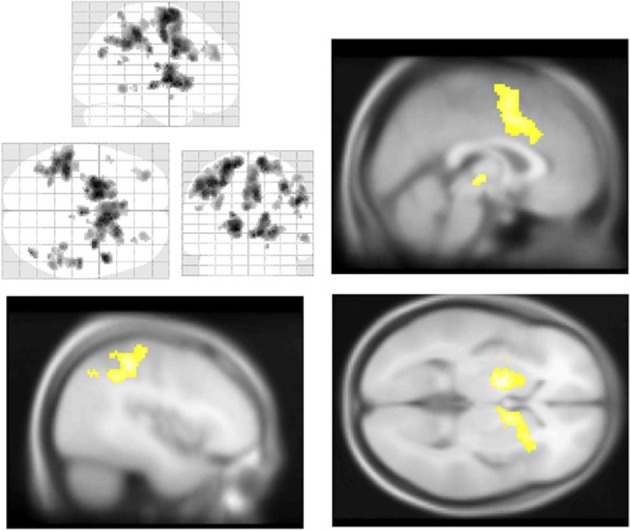
**Salience and default mode network BOLD responses to Aware > Unaware Errors**. During conscious error monitoring we observed significant activations throughout the salience and control networks, including mid-cingulate (**top right**), middle frontal, dorsolateral prefrontal, bilateral caudate (**bottom right**), right insula (**bottom right**), superior, and inferior parietal cortex (**bottom left**) activations. Note that in addition to common salience and error related regions, we observed bilateral inferior parietal responses to this contrast, i.e., co-activation of salience and DMN. Statistical parametric map (pFWE < 0.05, *k* threshold > 5 contiguous voxels) superimposed on SPM canonical anatomical image, average of 305 T1-weighted images. Top left “glass brain” displays activation extent in three dimensional space. Top right shown at MNI X coordinate = 1, bottom left at *X* = −43. **Bottom right** shown at *Z* = 1. See Table 5 for a complete list of foci.

Across participants, correct stops elicited significant BOLD activations throughout the canonical motor inhibition network, including bilateral anterior insula, superior parietal lobes, supplementary motor areas, and bilateral putamen (Hester et al., 2005; Wager et al., 2005; Verbruggen and Logan, 2008). Robust deactivations were observed in the DMN (dorsal mPFC and precuneus) as well as primary and secondary visual cortices (see Tables 3, 4, Figures 3, 4, for a complete summary of Stop-related responses). The Aware > Unaware contrast revealed significant activations in the salience and frontal-parietal attention networks (Seeley et al., 2007), including right anterior insula, thalamus, caudate nucleus, mid-cingulate cortex, middle frontal gyrus, and bilateral dorsolateral/rostral prefrontal cortex. Interestingly, we also observed significant activation of bilateral inferior parietal cortex, a region of the DMN, during aware errors. See Table 5 and Figure 5 for a complete summary of Aware > Unaware responses.

**Table 3:** **Brain regions displaying significant BOLD activations for correct stop trials compared to baseline**.

**Brain regions**	***Voxels k***	***p*FWE**	***T***	**MNI coordinates**		
				***x***	***y***	***z***
L Medial frontal gyrus	2560	<0.001	10.7	−6	−8	62
L Precentral		<0.001	10.68	−50	−10	54
R Supplementary		<0.001	7.44	10	−4	62
Motor						
R Insula	1057	<0.001	9.34	30	20	−6
R Anterior insula		<0.001	8.06	34	14	8
CSF near Putamen		<0.001	7.47	4	6	12
White matter near PCC	579	<0.001	8.46	6	−26	22
R Precentral	708	<0.001	7.6	48	−2	42
R Precentral (BA 6)		<0.001	7.53	56	−2	50
R Precentral (BA 9)		0.001	6.65	44	2	28
R Supramarginal	755	<0.001	7.5	60	−44	44
R Inferior parietal		<0.001	7.49	46	−40	46
R Inferior parietal		<0.001	7.33	56	−42	52
L Pallidum	1022	<0.001	7.2	−22	−6	16
L Anterior insula		0.001	7.01	−30	16	6
L Putamen		0.001	6.62	−20	4	2
L Inferior parietal	324	0.001	6.83	−44	−42	44
L Inferior parietal		0.007	6.04	−58	−46	44
L Inferior parietal		0.021	5.64	−52	−48	54
R Middle temporal	47	0.001	6.68	46	−40	6
L Thalamus	98	0.003	6.35	0	−12	−4
R Thalamus		0.024	5.57	14	−12	2
L Precentral	125	0.004	6.28	−42	0	26
Left rolandic		0.015	5.77	−48	0	18
operculum						
R Middle cingulate	28	0.007	6.02	10	16	38
L Inferior frontal	23	0.011	5.89	−48	32	32
R Fusiform	7	0.017	5.71	38	−48	−14
L Inferior parietal	12	0.02	5.65	−42	−46	30
R Middle temporal	9	0.023	5.6	64	−40	12
L Thalamus	6	0.025	5.56	−4	−2	−2

Cluster size k = number of contiguous voxels for that cluster. Contiguous sub-clusters shown beneath parent cluster. P-values peak-corrected for multiple comparisons, pFWE < 0.05.

**Table 4:** **Brain regions with significant BOLD activations to baseline > correct stops**.

**Brain region**	***Voxels k***	***p*FWE**	***T***	**MNI coordinates**		
				***x***	***y***	***z***
L Calcarine (BA 17)	5064	<0.001	10.73	−10	−96	16
L Calcarine (BA 17)		<0.001	10.51	−2	−92	10
R Calcarine (BA 17)		<0.001	10.03	6	−92	8
L Superior medial	932	<0.001	7.96	0	52	46
R Superior medial		<0.001	7.77	8	68	20
L Superior medial		<0.001	7.48	−2	66	18
R Supplementary	60	0.001	6.85	4	18	70
motor (BA 6)						
L Inferior frontal	34	0.01	5.9	−54	28	−2
L Middle temporal	6	0.021	5.64	−40	−58	20

Cluster size k = number of contiguous voxels for that cluster. Contiguous sub-clusters shown beneath parent cluster. P-values peak-corrected for multiple comparisons, pFWE < 0.05.

**Table 5:** **Brain regions with significant BOLD responses during aware > unaware errors**.

**Brain region**	***Voxels k***	***p*FWE**	***T***	**MNI coordinates**		
				***x***	***y***	***z***
L Pallidum	497	<0.001	7.68	−14	−4	2
L Caudate		0.004	6.53	−12	10	−4
L Putamen		0.009	6.2	−26	10	6
L Superior frontal	775	<0.001	7.52	−24	−6	64
L Superior		0.001	7.34	−16	−4	66
frontal (BA 6)						
L Superior		0.001	7.18	−28	−8	50
frontal (WM)						
R Inferior	126	0.001	7.35	50	4	30
frontal (BA 44)						
R Supplementary	1523	0.001	7.31	12	2	70
motor (BA6)						
R Middle		0.001	7.25	6	4	38
Cingulate						
R Superior		0.001	7.03	16	0	62
frontal						
R Caudate	615	0.001	7.3	10	4	−2
R Caudate (WM)		0.001	7.05	16	0	14
R Putamen		0.001	6.97	24	16	0
L Inferior	1267	0.001	7.28	−40	−34	48
parietal (BA 2)						
L Supramarginal		0.001	7.1	−48	−44	38
L Inferior parietal		0.001	7.1	−52	−34	46
R Precentral	100	0.001	7.08	34	−6	44
R Thalamus	67	0.001	7.06	4	−20	−4
R Supramarginal	324	0.002	6.86	54	−22	32
R Inferior parietal		0.005	6.45	54	−28	42
R Inferior parietal		0.005	6.44	52	−42	38
L Thalamus	20	0.002	6.76	−18	−14	16
R Middle temporal	49	0.004	6.54	44	−72	28
R Inferior temporal	57	0.008	6.25	56	−50	−6
L Middle frontal	136	0.008	6.24	−32	46	28
L Middle frontal		0.037	5.65	−38	34	32
R Middle temporal	13	0.012	6.09	48	−54	20
R Parahippocampal	6	0.014	6.04	18	0	−16
L Superior	20	0.014	6.03	−54	−48	22
temporal						
R Middle frontal	13	0.017	5.95	30	34	34
R Posterior	7	0.018	5.94	8	−32	36
Cingulate (in WM)						
R Superior frontal	47	0.018	5.94	26	42	26
R Inferior frontal (BA 44)	7	0.02	5.9	52	6	14
R Middle temporal	9	0.026	5.79	54	−36	−8
L Precentral (BA 44)	12	0.026	5.79	−50	8	38

Cluster size k = number of contiguous voxels for that cluster. Contiguous sub-clusters shown beneath parent cluster. P-values peak-corrected for multiple comparisons, pFWE < 0.05.

### fMRI—BOLD correlation with TUT reports

We found significant correlations between probe-related BOLD signal and TUT intensity reports in clusters located in the mPFC (*p*FWE = 0.038, *k* = 685), pCC (*p* = 0.004, *k* = 11), and superior parietal lobe (*p* = 0.004, *k* = 9) (See Figure 4, Table 6). Only the mPFC cluster survived correction for multiple comparisons. No significant clusters correlating positively with TUT were found. For comparison of spatial overlap, results from the TUT and Stop analyses were overlaid on a single image. This comparison suggested that while correct stops primarily elicited de-activations in a dorsal region of mPFC, TUT intensity predicted activity in a more rostral region (Figure 4). We found no significant correlations between probe-related BOLD signal and TUT stickiness.

**Table 6:** **Regions showing correlations between task-related BOLD and TUT rating**.

**Brain region**	***Voxels k***	***p*FWEc**	***pUnc***	**MNI coordinates**		
				***x***	***y***	***z***
L Superior medial	645	0.047	0.002	−12	60	8
R Superior medial			0.002	8	64	8
L Anterior cingulate			0.002	−8	44	6
^*^L Superior medial	89	0.776	0.004	−4	42	26
^*^L Inferior parietal	9	0.981	0.004	−46	−72	28
^*^L Posterior cingulate	9	0.981	0.006	−4	−56	22

Cluster size k = number of contiguous voxels for that cluster. Contiguous sub-clusters shown beneath parent cluster. P-values cluster corrected for multiple comparisons within default mode network mask, inclusion threshold P > 0.01 k threshold pFWEc < 0.05. Uncorrected p-values

(^*^) at inclusion threshold level shown for completeness. DMN mask generated using automated meta-analysis for “mPFC” on neurosynth.org, see Methods for details.

## Discussion

Consistent with previous fMRI work (Mason et al., 2007; Christoff et al., 2009; Stawarczyk et al., 2011), we found that during the EAT, intervals in which participants reported more frequent TUT predicted significant BOLD signal increases in the mPFC. We also found that correct stop trials were characterized by both deactivations in the dorsal mPFC and pCC, and increased activity in the motor inhibition and salience networks, suggesting an important interaction between these networks during cognitive control (Allen and Williams, 2011). Interestingly the portion of the mPFC deactivated by inhibition was in a non-overlapping portion of midline cortex, more dorsal to those voxels showing correlated responses to TUT intensity. While much of the research on task performance and the DMN has focused on their mutual antagonism, this finding may support some degree of functional segregation between self-generated thoughts and executive-related prefrontal inhibition. Additionally we found that during error awareness the inferior parietal cortex, a region of the DMN, was recruited along with common salience related regions such as insula and cingulate cortex. Coupled with our finding that mind-wandering variability predicts error monitoring performance, these results suggest that the relationship between task performance and self-generated thought may be more nuanced than mere antagonism.

Our results also inform our understanding of the link between TUT and task performance. We replicated the finding that overall levels of TUT can interfere with demanding tasks, potentially reflecting the role of TUT in facilitating perceptual decoupling (Smallwood, 2013). Consistent with prior studies we also found TUT reports were associated with greater mPFC activity (Mason et al., 2007; Christoff et al., 2009). Higher levels of variability in TUT were associated with a trend toward better stop performance, but more importantly, individuals who showed high variability in TUT more accurately detected mistakes made in the response inhibition task. Altogether, this pattern suggests that although mind-wandering has negative consequences for task performance, individuals who balance states of self-generated and task related experiences are relatively more effective at monitoring their performance. Importantly, a simple demand characteristic explanation would not predict these results as participants were motivated by financial rewards to perform the task as well as possible. As self-generated thought has both costs and benefits (McVay et al., 2008; Smallwood et al., 2008a; Baird et al., 2011; Mrazek et al., 2012; Smallwood and Andrews-Hanna, 2013; Smallwood et al., 2013a), it is possible that the association between metacognition and greater variability in mental states reflects an increased ability to balance self-generated thought and external perceptual processes, optimizing task performance over both immediate and more temporally extended events.

In general, behavioral variability reflects the sensitivity of cognition to fluctuating task demands, and can produce positive or negative outcomes depending on behavioral context (Lutz et al., 2002). Conscious error awareness depends upon integrating interoceptive error cues with visual-motor control signals (Sridharan et al., 2008; Ullsperger et al., 2010; Klein et al., 2013). Our finding that individual differences in TUT variability relate to error monitoring performance may thus depend upon the individual capacity to flexibly switch between and integrate across different information sources. Alternatively, our data may simply indicate that both online performance monitoring and flexibility in the contents of conscious thought both depend on a single domain general metacognitive process. However, this later interpretation is inconsistent with prior studies indicating that the correlation between metacognitive accuracy for memory and perception is unreliable and that success in both domains both depend on distinct resting neural networks (Baird et al., 2013).

Regardless of the specific relationship, our data extends the role of metacognition in enhancing the flexibility of conscious thought (Flavell, 1979; Shimamura, 2000). One speculative implication of this result is that by utilizing metacognition to reduce perseveration on either internal or external information, an individual may be able to exploit the benefits of self-generated thought while minimizing the costs as far as possible. One possible mechanism supporting such a benefit may be an increased ability to regulate the context and content in which self-generated thought occurs (Smallwood and Andrews-Hanna, 2013). More generally our data suggest that the role of metacognitive monitoring in facilitating TUT may explain why the mPFC, a region implicated in metacognition (Schmitz et al., 2004; Fleming et al., 2012; Frith and Frith, 2012), is also active during self-generated thought. Plausibly, the mPFC could allow an individual to reflect upon the contents of their self-generated thoughts and so benefit from this memory driven mode of thought to make progress on their ongoing behavioral goals. Future research should explore the possibility that certain forms of self-generated thought entail metacognitive processing in coordinating the occurrence or the content of the experience.

Although we advance an interpretation of TUT variance as relating to metacognition, it must be noted that there is emerging evidence for a functional dissociation of reflective metacognition and online error monitoring processes (Fleming et al., 2012). While the former is typically thought to involve top-down conscious judgments of the reliability of a particular source of information, error awareness has increasingly been shown to involve distinct functional systems, particularly the salience network, e.g., anterior insula and rostral cingulate. Both reflective metacognition and error monitoring are thought to contribute to such self-evaluations and are impaired by lesions to the PFC and anterior cingulate cortex (Hoerold et al., 2012). However, signal-theoretic models suggest that error awareness may contribute more directly to an interoceptive sense of uncertainty or doubt, or a graded subliminal awareness of errors (Fleming et al., 2012; Charles et al., 2013). Thus, an interesting and unresolved question for future research is whether meta-cognitive confidence in TUT ratings might predict stop-accuracy, as predicted by the meta-awareness hypothesis (Schooler, 2002; Maniscalco and Lau, 2012).

Our results also have implications for understanding the component process view of the DMN (Andrews-Hanna et al., 2010; Andrews-Hanna, 2012). We found that the intensity of self-reported TUTs predicted activations in the mPFC, and that successful stops de-activated a more dorsal region of the mPFC. Thus, our results suggest a dissociation between elements of the DMN: Both the pCC and dorsal areas of the mPFC are inhibited when individuals engage in cognitive control, whereas more rostral regions of the mPFC are engaged during self-generated thought. Consistent with the distinction our data suggests, a graph theoretical analysis of the DMN implicates ventral regions of mPFC in the midline core of the system, while dorsal regions of the mPFC participates in what is known as the dorsal medial pre-frontal subsystem (Andrews-Hanna et al., 2010). Based on our data we speculate that more rostral-prefrontal regions of mPFC may be especially important in the self-generation of mental contents that are unrelated to ongoing task performance, an observation which is consistent with evidence that this brain region is linked to self-referent information processing (Mitchell et al., 2005; Mitchell, 2009). In contrast, dorsal regions of mPFC were deactivated when cognitive control was employed on task relevant information, supporting suggestions that this region may play a more general role in states of decoupled processing regardless of whether they are based on personally relevant information (see also Smallwood et al., 2013b).

## Future directions

In the present design we attempted to control participant motivation through financial reward; it is possible that our motivation manipulation interacted with self-reports, although we specifically instructed participants to report their honest experience. Indeed, we observed activations in reward-related areas including the caudate nucleus and putamen in our error awareness and stop contrasts (Schultz, 2000; Haruno and Kawato, 2006). Future research may benefit from shorter task intervals in a behavioral setting, to establish the role of motivational reward in reports of mind-wandering behavior (see for example Mrazek et al., 2012). Our results also suggest that TUT accounted for a significant amount of variability in the EAT. Previous research has implicated disrupted error awareness in ADHD and cocaine abuse (Hester et al., 2007; O'Connell et al., 2009); our findings suggest that such disruptions could be related to reduced variability of mind-wandering during the EAT and/or increased overall TUT, reflecting cognitive rigidity.

We also attempted to apply a subjective distinction between the intensity or subjective frequency of TUTs and their “stickiness,” to generate self-reports capturing unique aspects of phenomenological experience, effectively “front-loading” phenomenological intuition into our experimental design (Gallagher, 2003). Although we attempted to create a distinction between the subjective frequency of TUTs and their attention-capturing nature, our data suggest a large degree of colinearity in these measures. This null-finding raises the possibility that a more prolonged familiarization of participants with subtle subjective categories is required to measure them empirically (Lutz et al., 2002). However, because variability in the experience of TUT showed a pattern suggestive of better performance and superior monitoring, it is possible that individuals who are high on mean levels of TUT and lack variance reflect a population for whom self-generated thoughts are especially sticky and hence problematic. While we note that a limitation of the present design is a lack of validation for the stickiness measure, future methodological research should consider this possibility.

## Conclusion

In conclusion, our findings confirm previous work suggesting that TUTs interfere with task performance under demanding task conditions. In addition, we found novel evidence that variability in mind-wandering experience related to greater metacognitive ability, suggesting a role for online monitoring in ensuring flexibility in the manner that attention is deployed on both external and self-generated sources of information. We observed activations of both default mode and salience networks during error monitoring, a finding in line with the observation that particular aspects of mind-wandering are related to self-monitoring. We also found that ventral regions of the mPFC increased activity as TUT increased, while more dorsal regions were deactivated when individuals engaged cognitive control, a finding broadly in line with a component process view of the DMN. Given these results we recommend that a time series analysis of subjective variability with continuous self-report measures, or investigation of second-order confidence in TUT ratings, may reveal further granularity in the experience of mind-wandering and related contributions to behavioral performance. Such approaches may prove important in determining the extent to which individuals regulate the balance of conscious thought so as to maximize the benefits of self-generated thought, while simultaneously limiting its costs.

### Conflict of interest statement

The authors declare that the research was conducted in the absence of any commercial or financial relationships that could be construed as a potential conflict of interest.

## References

[B1] Allen M. Williams G. (2011). Consciousness, plasticity, and connectomics: the role of intersubjectivity in human cognition. Front. Psychol. 2:20 10.3389/fpsyg.2011.00020 21687435 PMC3110420

[B2] Amodio D. M. Frith C. D. (2006). Meeting of minds: the medial frontal cortex and social cognition. Nat. Rev. Neurosci. 7, 268–277 10.1038/nrn1884 16552413

[B3] Andrews-Hanna J. R. (2012). The brain's default network and its adaptive role in internal mentation. Neuroscientist 18, 251–270 10.1177/1073858411403316 21677128 PMC3553600

[B4] Andrews-Hanna J. R. Reidler J. S. Sepulcre J. Poulin R. Buckner R. L. (2010). Functional-anatomic fractionation of the brain's default network. Neuron 65, 550–562 10.1016/j.neuron.2010.02.005 20188659 PMC2848443

[B5] Anticevic A. Repovs G. Shulman G. L. Barch D. M. (2010). When less is more: TPJ and default network deactivation during encoding predicts working memory performance. Neuroimage 49, 2638–2648 10.1016/j.neuroimage.2009.11.008 19913622 PMC3226712

[B6] Ashburner J. Friston K. J. (1999). Nonlinear spatial normalization using basis functions. Hum. Brain Mapp. 7, 254–266 10.1002/(SICI)1097-0193(1999)7:4<254::AID-BM4>3.0.CO;2-G 10408769 PMC6873340

[B7] Baird B. Smallwood J. Gorgolewski K. Marguiles D. (2013). Medial and lateral networks in anterior prefrontal cortex support metacognition ability for memory and perception. J. Neurosci. 33, 16657–16665 10.1523/JNEUROSCI.0786-13.2013 24133268 PMC6618531

[B8] Baird B. Smallwood J. Mrazek M. D. Kam J. W. Y. Franklin M. S. Schooler J. W. (2012). Inspired by distraction: mind wandering facilitates creative incubation. Psychol. Sci. 23, 1117–1122 10.1177/0956797612446024 22941876

[B9] Baird B. Smallwood J. Schooler J. W. (2011). Back to the future: autobiographical planning and the functionality of mind-wandering. Conscious. Cogn. 20, 1604–1611 10.1016/j.concog.2011.08.007 21917482

[B10] Buckner R. L. Carroll D. C. (2007). Self-projection and the brain. Trends Cogn. Sci. 11, 49–57 10.1016/j.tics.2006.11.004 17188554

[B11] Burgess P. W. Gilbert S. J. Dumontheil I. (2007). Function and localization within rostral prefrontal cortex (area 10). Philos. Trans. R. Soc. B Biol. Sci. 362, 887–899 10.1098/rstb.2007.2095 17403644 PMC2430004

[B12] Chang C. Cunningham J. P. Glover G. H. (2009). Influence of heart rate on the BOLD signal: the cardiac response function. Neuroimage 44, 857–869 10.1016/j.neuroimage.2008.09.029 18951982 PMC2677820

[B13] Chang C. Glover G. H. (2009). Relationship between respiration, end-tidal CO2, and BOLD signals in resting-state fMRI. Neuroimage 47, 1381–1393 10.1016/j.neuroimage.2009.04.048 19393322 PMC2721281

[B14] Charles L. Opstal F. van Marti S. Dehaene S. (2013). Distinct brain mechanisms for conscious versus subliminal error detection. Neuroimage 73, 80–94 10.1016/j.neuroimage.2013.01.054 23380166 PMC5635965

[B15] Christoff K. Gordon A. M. Smallwood J. Smith R. Schooler J. W. (2009). Experience sampling during fMRI reveals default network and executive system contributions to mind wandering. Proc. Natl. Acad. Sci. U.S.A. 106, 8719–8724 10.1073/pnas.0900234106 19433790 PMC2689035

[B16] Dumontheil I. Gilbert S. J. Frith C. D. Burgess P. W. (2010a). Recruitment of lateral rostral prefrontal cortex in spontaneous and task-related thoughts. Q. J. Exp. Psychol. 63, 1740–1756 10.1080/17470210903538114 20221947

[B17] Dumontheil I. Hassan B. Gilbert S. J. Blakemore S.-J. (2010b). Development of the selection and manipulation of self-generated thoughts in adolescence. J. Neurosci. 30, 7664–7671 10.1523/JNEUROSCI.1375-10.2010 20519541 PMC6632361

[B18] Epstein J. N. Langberg J. M. Rosen P. J. Graham A. Narad M. E. Antonini T. N. (2011). Evidence for higher reaction time variability for children with ADHD on a range of cognitive tasks including reward and event rate manipulations. Neuropsychology 25, 427 10.1037/a0022155 21463041 PMC3522094

[B19] Farrar D. E. Glauber R. R. (1967). Multicollinearity in regression analysis: the problem revisited. Rev. Econ. Stat. 49, 92–107 10.2307/1937887

[B20] Flavell J. H. (1979). Metacognition and cognitive monitoring. Am. Psychol. 34, 906–911 10.1037/0003-066X.34.10.906

[B21] Fleming S. M. Dolan R. J. Frith C. D. (2012). Metacognition: computation, biology and function. Philos. Trans. R. Soc. Lond. B. Biol. Sci. 367, 1280–1286 10.1098/rstb.2012.0021 22492746 PMC3318771

[B22] Fleming S. M. Weil R. S. Nagy Z. Dolan R. J. Rees G. (2010). Relating introspective accuracy to individual differences in brain structure. Science 329, 1541–1543 10.1126/science.1191883 20847276 PMC3173849

[B23] Fox M. D. Snyder A. Z. Vincent J. L. Corbetta M. Van Essen D. C. Raichle M. E. (2005). The human brain is intrinsically organized into dynamic, anticorrelated functional networks. Proc. Natl. Acad. Sci. U. S. A. 102, 9673–9678 10.1073/pnas.0504136102 15976020 PMC1157105

[B24] Friston K. Ashburner J. Frith C. D. Poline J. Heather J. D. Frackowiak R. S. J. (1995). Spatial registration and normalization of images. Hum. Brain Mapp. 3, 165–189 10.1002/hbm.460030303

[B25] Friston K. J. Penny W. D. Ashburner J. Kiebel S. J. Nichols T. E. (2006). Statistical parametric mapping: the analysis of functional brain images. London: Academic Press ISBN: 9780123725608

[B26] Friston K. J. Holmes A. P. Worsley K. J. Poline J.-P. Frith C. D. Frackowiak R. S. J. (1994). Statistical parametric maps in functional imaging: a general linear approach. Hum. Brain Mapp. 2, 189–210 10.1002/hbm.460020402

[B27] Friston K. J. Josephs O. Zarahn E. Holmes A. P. Rouquette S. Poline J. (2000). To smooth or not to smooth? Bias and efficiency in fMRI time-series analysis. Neuroimage 12, 196–208 10.1006/nimg.2000.0609 10913325

[B28] Friston K. J. Williams S. Howard R. Frackowiak R. S. J. Turner R. (1996). Movement−related effects in fMRI time−series. Magn. Reson. Med. 35, 346–355 10.1002/mrm.1910350312 8699946

[B29] Frith C. D. Frith U. (2012). Mechanisms of social cognition. Annu. Rev. Psychol. 63, 287–313 10.1146/annurev-psych-120710-100449 21838544

[B30] Gallagher S. (2003). Phenomenology and experimental design toward a phenomenologically enlightened experimental science. J. Conscious. Stud. 10, 9–10

[B31] Glover G. H. Li T. Q. Ress D. (2000). Image-based method for retrospective correction of physiological motion effects in fMRI: RETROICOR. Magn. Reson. Med. 44, 162–167 10.1002/1522-2594(200007)44:1<162::AID-MRM23>3.3.CO;2-5 10893535

[B32] Greicius M. D. Krasnow B. Reiss A. L. Menon V. (2003). Functional connectivity in the resting brain: a network analysis of the default mode hypothesis. Proc. Natl. Acad. Sci. U.S.A. 100, 253–258 10.1073/pnas.0135058100 12506194 PMC140943

[B33] Hampson M. Driesen N. R. Skudlarski P. Gore J. C. Constable R. T. (2006). Brain connectivity related to working memory performance. J. Neurosci. 26, 13338–13343 10.1523/JNEUROSCI.3408-06.2006 17182784 PMC2677699

[B34] Haruno M. Kawato M. (2006). Different neural correlates of reward expectation and reward expectation error in the putamen and caudate nucleus during stimulus-action-reward association learning. J. Neurophysiol. 95, 948–959 10.1152/jn.00382.2005 16192338

[B35] Hayasaka S. Phan K. L. Liberzon I. Worsley K. J. Nichols T. E. (2004). Nonstationary cluster-size inference with random field and permutation methods. Neuroimage 22, 676–687 10.1016/j.neuroimage.2004.01.041 15193596

[B36] Hester R. Foxe J. J. Molholm S. Shpaner M. Garavan H. (2005). Neural mechanisms involved in error processing: a comparison of errors made with and without awareness. Neuroimage 27, 602–608 10.1016/j.neuroimage.2005.04.035 16024258

[B37] Hester R. Nandam L. S. O'Connell R. G. Wagner J. Strudwick M. Nathan P. J. (2012). Neurochemical enhancement of conscious error awareness. J. Neurosci. 32, 2619–2627 10.1523/JNEUROSCI.4052-11.2012 22357846 PMC6621889

[B38] Hester R. Nestor L. Garavan H. (2009). Impaired error awareness and anterior cingulate cortex hypoactivity in chronic cannabis users. Neuropsychopharmacology 34, 2450–2458 10.1038/npp.2009.67 19553917 PMC2743772

[B39] Hester R. Simoes-Franklin C. Garavan H. (2007). Post-error behavior in active cocaine users: poor awareness of errors in the presence of intact performance adjustments. Neuropsychopharmacology 32, 1974–1984 10.1038/sj.npp.1301326 17268406

[B40] Hoerold D. Pender N. P. Robertson I. H. (2012). Metacognitive and online error awareness deficits after prefrontal cortex lesions. Neuropsychologia 51, 385–391 10.1016/j.neuropsychologia.2012.11.019 23196146

[B41] Hultsch D. F. MacDonald S. W. S. Dixon R. A. (2002). Variability in reaction time performance of younger and older adults. J. Gerontol. Ser. B Psychol. Sci. Soc. Sci. 57, P101–P115 10.1093/geronb/57.2.P101 11867658

[B42] Jack A. I. Roepstorff A. (2002). Introspection and cognitive brain mapping: from stimulus–response to script–report. Trends Cogn. Sci. 6, 333–339 10.1016/S1364-6613(02)01941-1 12140083

[B43] Jensen C. G. Vangkilde S. Frokjaer V. Hasselbalch S. G. (2012). Mindfulness training affects attention–or is it attentional effort. J. Exp. Psychol. Gen 141, 106–123 10.1037/a0024931 21910559

[B44] Killingsworth M. A. Gilbert D. T. (2010). A wandering mind is an unhappy mind. Science 330, 932 10.1126/science.1192439 21071660

[B45] Klein T. a Ullsperger M. Danielmeier C. (2013). Error awareness and the insula: links to neurological and psychiatric diseases. Front. Hum. Neurosci. 7:14 10.3389/fnhum.2013.00014 23382714 PMC3563042

[B46] Koster E. H. W. De Lissnyder E. Derakshan N. De Raedt R. (2011). Understanding depressive rumination from a cognitive science perspective: the impaired disengagement hypothesis. Clin. Psychol. Rev. 31, 138–145 10.1016/j.cpr.2010.08.005 20817334

[B47] Larson G. E. Alderton D. L. (1990). Reaction time variability and intelligence: a “worst performance” analysis of individual differences. Intelligence 14, 309–325 10.1016/0160-2896(90)90021-K

[B48] Leth-Steensen C. King Elbaz Z. Douglas V. I. (2000). Mean response times, variability, and skew in the responding of ADHD children: a response time distributional approach. Acta Psychol. (Amst). 104, 167–190 10.1016/S0001-6918(00)00019-6 10900704

[B49] Lund T. E. Madsen K. H. Sidaros K. Luo W.-L. Nichols T. E. (2006). Non-white noise in fMRI: does modelling have an impact? Neuroimage 29, 54–66 10.1016/j.neuroimage.2005.07.005 16099175

[B50] Lutz A. Lachaux J.-P. Martinerie J. Varela F. J. (2002). Guiding the study of brain dynamics by using first-person data: synchrony patterns correlate with ongoing conscious states during a simple visual task. Proc. Natl. Acad. Sci. U.S.A. 99, 1586–1591 10.1073/pnas.032658199 11805299 PMC122234

[B51] Lutz A. Slagter H. A. Rawlings N. B. Francis A. D. Greischar L. L. Davidson R. J. (2009). Mental training enhances attentional stability: neural and behavioral evidence. J. Neurosci. 29, 13418–16474 10.1523/JNEUROSCI.1614-09.2009 19846729 PMC2789281

[B52] Maldjian J. A. Laurienti P. J. Burdette J. H. (2004). Precentral gyrus discrepancy in electronic versions of the Talairach atlas. Neuroimage 21, 450–455 10.1016/j.neuroimage.2003.09.032 14741682

[B53] Maldjian J. A. Laurienti P. J. Kraft R. A. Burdette J. H. (2003). An automated method for neuroanatomic and cytoarchitectonic atlas-based interrogation of fMRI data sets. Neuroimage 19, 1233 10.1016/S1053-8119(03)00169-1 12880848

[B54] Maniscalco B. Lau H. (2012). A signal detection theoretic approach for estimating metacognitive sensitivity from confidence ratings. Conscious. Cogn. 21, 422–430 10.1016/j.concog.2011.09.021 22071269

[B55] Marchetti I. Koster E. H. W. De Raedt R. (2012). Mindwandering heightens the accessibility of negative relative to positive thought. Conscious. Cogn. 21, 1517–1525 10.1016/j.concog.2012.05.013 22726693

[B56] Mason M. F. Norton M. I. Van Horn J. D. Wegner D. M. Grafton S. T. Macrae C. N. (2007). Wandering minds: the default network and stimulus-independent thought. Science 315, 393–395 10.1126/science.1131295 17234951 PMC1821121

[B57] McVay J. C. Knouse L. Mitchell J. Brown L. Kane M. J. Kwapil T. R. (2008). Impaired conductors in the train of thought? Mind wandering in attention deficit/hyperactivity disorder, in Poster Presented at the Annual Meeting of Association for Psychological Science, Chicago, IL.

[B58a] Mitchell J. P. Banaji M. R. Macrae C. N. (2005). General and specific contributions of the medial prefrontal cortex to knowledge about mental states. Neuroimage 28, 757–8119 10.1016/j.neuroimage.2005.03.011 16325141

[B58] Mitchell J. P. (2009). Social psychology as a natural kind. Trends Cogn. Sci. 13, 246–251 10.1016/j.tics.2009.03.008 19427258 PMC2935896

[B59] Mrazek M. D. Smallwood J. Franklin M. S. Chin J. M. Baird B. Schooler J. W. (2012). The role of mind-wandering in measurements of general aptitude. J. Exp. Psychol. Gen. 141, 788–798 10.1037/a0027968 22468669

[B60] Murphy K. Birn R. M. Handwerker D. A Jones T. B. Bandettini P. A. (2009). The impact of global signal regression on resting state correlations: are anti-correlated networks introduced? Neuroimage 44, 893–905 10.1016/j.neuroimage.2008.09.036 18976716 PMC2750906

[B61] O'Connell R. G. Bellgrove M. A. Dockree P. M. Lau A. Hester R. Garavan H. (2009). The neural correlates of deficient error awareness in attention-deficit hyperactivity disorder (ADHD). Neuropsychologia 47, 1149–1159 10.1016/j.neuroimage.2008.09.036 19350709

[B62] Rabbitt P. (2002). Consciousness is slower than you think. Q. J. Exp. Psychol. A. 55, 1081–1092 10.1080/02724980244000080 12420985

[B63] Rabbitt P. M. (1966). Errors and error correction in choice-response tasks. J. Exp. Psychol. 71, 264–272 10.1037/h0022853 5948188

[B65] Schad D. J. Nuthmann A. Engbert R. (2012). Your mind wanders weakly, your mind wanders deeply: objective measures reveal mindless reading at different levels. Cognition 125, 179–194 10.1016/j.cognition.2012.07.004 22857818

[B66] Schmitz T. W. Kawahara-Baccus T. N. Johnson S. C. (2004). Metacognitive evaluation, self-relevance, and the right prefrontal cortex. Neuroimage 22, 941–947 10.1016/j.neuroimage.2004.02.018 15193625

[B67] Schooler J. W. (2002). Re-representing consciousness: dissociations between experience and meta-consciousness. Trends Cogn. Sci. 6, 339–344 10.1016/S1364-6613(02)01949-6 12140084

[B68] Schultz W. (2000). Multiple reward signals in the brain. Nat. Rev. Neurosci. 1, 199–207 10.1038/35044563 11257908

[B69] Seeley W. W. Menon V. Schatzberg A. F. Keller J. Glover G. H. Kenna H. (2007). Dissociable intrinsic connectivity networks for salience processing and executive control. J. Neurosci. 27, 2349–2356 10.1523/JNEUROSCI.5587-06.2007 17329432 PMC2680293

[B70] Shalgi S. O'Connell R. G. Deouell L. Y. Robertson I. H. (2007). Absent minded but accurate: delaying responses increases accuracy but decreases error awareness. Exp. Brain Res. 182, 119–124 10.1007/S00221-007-1054-5 17634930

[B71] Shiels K. Tamm L. Epstein J. N. (2012). Deficient post-error slowing in children with ADHD is limited to the inattentive subtype. J. Int. Neuropsychol. Soc. 18, 612–617 10.1017/S1355617712000082 22390841 PMC3348350

[B72] Shimamura A. P. (2000). Toward a cognitive neuroscience of metacognition. Conscious. Cogn. 9, 313–323 10.1006/ccog.2000.0450 10924251

[B73] Smallwood J. (2013). Distinguishing how from why the mind wanders: a process–occurrence framework for self-generated mental activity. Psychol. Bull. 139, 519 10.1037/a0030010 23607430

[B74] Smallwood J. Andrews-Hanna J. (2013). Not all minds that wander are lost: the importance of a balanced perspective on the mind-wandering state. Front. Psychol. 4:441 10.3389/fpsyg.2013.00441 23966961 PMC3744871

[B75] Smallwood J. Beach E. Schooler J. W. Handy T. C. (2008a). Going AWOL in the brain: mind wandering reduces cortical analysis of external events. J. Cogn. Neurosci. 20, 458–469 10.1162/jocn.2008.20037 18004943

[B76] Smallwood J. McSpadden M. Luus B. Schooler J. (2008b). Segmenting the stream of consciousness: the psychological correlates of temporal structures in the time series data of a continuous performance task. Brain Cogn. 66, 50–56 10.1016/j.bandc.2007.05.004 17614178

[B77] Smallwood J. Fishman D. J. Schooler J. W. (2007a). Counting the cost of an absent mind: mind wandering as an underrecognized influence on educational performance. Psychon. Bull. Rev. 14, 230–236 10.3758/BF03194057 17694906

[B78] Smallwood J. O'Connor R. C. Sudbery M. V. Obonsawin M. (2007b). Mind-wandering and dysphoria. Cogn. Emot. 21, 816–842 10.1080/02699930600911531

[B79] Smallwood J. Fitzgerald A. Miles L. K. Phillips L. H. (2009). Shifting moods, wandering minds: negative moods lead the mind to wander. Emotion 9, 271–276 10.1037/a0014855 19348539

[B80] Smallwood J. Ruby F. J. M. Singer T. (2013a). Letting go of the present: mind-wandering is associated with reduced delay discounting. Conscious. Cogn. 22, 1–7 10.1016/j.concog.2012.10.007 23178292

[B81] Smallwood J. Tipper C. Brown K. Baird B. Engen H. Michaels J., R (2013b). Escaping the here and now: evidence for a role of the default mode network in perceptually decoupled thought. Neuroimage 69, 120–125 10.1016/j.neuroimage.2012.12.012 23261640

[B82] Smallwood J. Schooler J. W. Turk D. J. Cunningham S. J. Burns P. Macrae C. N. (2011). Self-reflection and the temporal focus of the wandering mind. Conscious. Cogn. 20, 1120–1126 10.1016/j.concog.2010.12.017 21277803

[B83] Spreng R. N. Stevens W. D. Chamberlain J. P. Gilmore A. W. Schacter D. L. (2010). Default network activity, coupled with the frontoparietal control network, supports goal-directed cognition. Neuroimage 53, 303–317 10.1016/j.neuroimage.2010.06.016 20600998 PMC2914129

[B84] Sridharan D. Levitin D. J. Menon V. (2008). A critical role for the right fronto-insular cortex in switching between central-executive and default-mode networks. Proc. Natl. Acad. Sci. U.S.A. 105, 12569–12574 10.1073/pnas.0800005105 18723676 PMC2527952

[B85] Stawarczyk D. Majerus S. Maquet P. D'Argembeau A. (2011). Neural correlates of ongoing conscious experience: both task-unrelatedness and stimulus-independence are related to default network activity. PLoS ONE 6:e16997 10.1371/journal.pone.0016997 21347270 PMC3038939

[B86] Stuss D. T. Pogue J. Buckle L. Bondar J. (1994). Characterization of stability of performance in patients with traumatic brain injury: variability and consistency on reaction time tests. Neuropsychology 8, 316 10.1037/0894-4105.8.3.316

[B87] Ullsperger M. Harsay H. a Wessel J. R. Ridderinkhof K. R. (2010). Conscious perception of errors and its relation to the anterior insula. Brain Struct. Funct. 214, 629–643 10.1007/s00429-010-0261-1 20512371 PMC2886909

[B88] Van Vugt M. K. Hitchcock P. Shahar B. Britton W. (2012). The effects of mindfulness-based cognitive therapy on affective memory recall dynamics in depression: a mechanistic model of rumination. Front. Hum. Neurosci. 6:257 10.3389/fnhum.2012.00257 23049507 PMC3446543

[B89] Varela F. Lachaux J. Rodriguez E. Martinerie J. (2001). The brainweb: large-scale phase integration. Nat. Rev. Neurosci. 2, 229–239 10.1038/35067550 11283746

[B90] Vaurio R. G. Simmonds D. J. Mostofsky S. H. (2009). Increased intra-individual reaction time variability in attention-deficit/hyperactivity disorder across response inhibition tasks with different cognitive demands. Neuropsychologia 47, 2389–2396 10.1016/j.neuropsychologia.2009.01.022 19552927 PMC4766847

[B91] Verbruggen F. Logan G. D. (2008). Response inhibition in the stop-signal paradigm. Trends Cogn. Sci. 12, 418–424 10.1016/j.tics.2008.07.005 18799345 PMC2709177

[B92] Wager T. D. Sylvester C.-Y. C. Lacey S. C. Nee D. E. Franklin M. Jonides J. (2005). Common and unique components of response inhibition revealed by fMRI. Neuroimage 27, 323–340 10.1016/j.neuroimage.2005.01.054 16019232

[B93] Weyandt L. L. Iwaszuk W. Fulton K. Ollerton M. Beatty N. Fouts H. (2003). The internal restlessness scale performance of college students with and without ADHD. J. Learn. Disabil. 36, 382–389 10.1177/00222194030360040801 15490909

[B94] Worsley K. J. Friston K. J. (1995). Analysis of fMRI time-series revisited–again. Neuroimage 2, 173–181 10.1006/nimg.1995.1023 9343600

[B95] Worsley K. J. Marrett S. Neelin P. Vandal A. C. Friston K. J. Evans A. C. (1996). A unified statistical approach for determining significant signals in images of cerebral activation. Hum. Brain Mapp. 4, 58–73 10.1002/(SICI)1097-0193(1996)4:1<58::AID-HBM4>3.0.CO;2-0 20408186

[B96] Yarkoni T. Poldrack R. A. Nichols T. E. Van Essen D. C. Wager T. D. (2011). Large-scale automated synthesis of human functional neuroimaging data. Nat. Methods 8, 665–670 10.1038/nmeth.1635 21706013 PMC3146590

